# Stage-independent biomarkers for Alzheimer’s disease from the living retina: an animal study

**DOI:** 10.1038/s41598-022-18113-y

**Published:** 2022-08-11

**Authors:** Hugo Ferreira, Pedro Serranho, Pedro Guimarães, Rita Trindade, João Martins, Paula I. Moreira, António Francisco Ambrósio, Miguel Castelo-Branco, Rui Bernardes

**Affiliations:** 1 Coimbra Institute for Biomedical Imaging and Translational Research (CIBIT), Institute for Nuclear Sciences Applied to Health (ICNAS), University of Coimbra, Azinhaga de Santa Comba, 3000-548 Coimbra, Portugal; 2 Department of Sciences and Technology, Universidade Aberta, Rua da Escola Politécnica, n.º 147, 1269-001 Lisboa, Portugal; 3 Coimbra Institute for Clinical and Biomedical Research (iCBR), Faculty of Medicine (FMUC), University of Coimbra, Azinhaga de Santa Comba, 3000-548 Coimbra, Portugal; 4 Center for Innovative Biomedicine and Biotechnology (CIBB), University of Coimbra, Azinhaga de Santa Comba, 3000-548 Coimbra, Portugal; 5 Clinical Academic Center of Coimbra (CACC), Faculty of Medicine (FMUC), University of Coimbra, Azinhaga de Santa Comba, 3000-548 Coimbra, Portugal; 6 Laboratory of Physiology, Faculty of Medicine (FMUC), University of Coimbra, Azinhaga de Santa Comba, 3000-548 Coimbra, Portugal; 7 Center for Neuroscience and Cell Biology (CNC), University of Coimbra, Azinhaga de Santa Comba, 3000-548 Coimbra, Portugal

**Keywords:** Diagnostic markers, Predictive markers, Prognostic markers, Alzheimer's disease, Neurodegeneration, Biomedical engineering

## Abstract

The early diagnosis of neurodegenerative disorders is still an open issue despite the many efforts to address this problem. In particular, Alzheimer’s disease (AD) remains undiagnosed for over a decade before the first symptoms. Optical coherence tomography (OCT) is now common and widely available and has been used to image the retina of AD patients and healthy controls to search for biomarkers of neurodegeneration. However, early diagnosis tools would need to rely on images of patients in early AD stages, which are not available due to late diagnosis. To shed light on how to overcome this obstacle, we resort to 57 wild-type mice and 57 triple-transgenic mouse model of AD to train a network with mice aged 3, 4, and 8 months and classify mice at the ages of 1, 2, and 12 months. To this end, we computed fundus images from OCT data and trained a convolution neural network (CNN) to classify those into the wild-type or transgenic group. CNN performance accuracy ranged from 80 to 88% for mice out of the training group’s age, raising the possibility of diagnosing AD before the first symptoms through the non-invasive imaging of the retina.

## Introduction

Despite the abundant number of studies and distinct diagnostic approaches followed, it is still widely accepted that even with the current state-of-the-art techniques, Alzheimer’s disease (AD) remains broadly undiagnosed for over a decade before first suspicions^1,2^ because screening the entire asymptomatic population is not feasible^1^.

The striking impact of this delayed diagnosis is the loss of the time window where intervention could have a substantial effect by halting or delaying the disease progression. Therefore, a fundamentally distinct approach is of paramount importance; due to the general increase in life expectancy, AD affects an increasing number of people worldwide, with substantial social and economic impact and a significant burden to patients, family members, and caregivers^2^.

In the last decade, the use of artificial intelligence/machine learning (ML) systems for disease diagnosis exploded because of the advances in computing power and artificial neural networks (NN), especially on the development and use of convolution neural networks (CNN)^3,4^. These, when given the proper dataset and adequate training, can analyse and classify images. Indeed, the use of CNNs is now widespread in many fields, medicine included. Its use has been proven efficient, achieving impressive performance in disease diagnosis and identifying lesions within a wealth of medical image modalities^4–14^.

Despite the many and clear advantages of NNs, typically, NNs are trained to discriminate between the examples provided but are not forced to learn features that can be applied out of the conditions of the training set. Consequently, it can only be expectable that these systems allow overcoming the lack of experts in a particular field by processing/analyzing significant amounts of data, make the diagnosis faster or cheaper, or all the above. However, a NN is typically limited by the training data (ground truth); concerning medical data, early diagnosis is out of reach.

The use of the retina and animal models of disease to study AD is well-grounded because of the hurdles in studying neurodegenerative disorders in humans based on brain imaging. The retina is the only part of the central nervous system (CNS) readily available through non-invasive optical means^15^ and shares its embryonic origin with the brain^16^. Thus, it has been used as a source of information on the changes unfolding in the CNS and as a surrogate for changes unfolding in the brain. Another obstacle in studying neurodegenerative disorders is the time scale in which these changes unfold. As mentioned above, AD is considered to develop for many years before possible diagnosis, with all associated consequences; unknown changes at the onset of the disease and the impossibility to intervene when changes are still minute and would have a more significant impact on disease progression.

The above reasons led us to design and perform the present study in which we aim to answer two scientific questions in the context of discrimination between the retinas of wild-type and the triple-transgenic (3xTg-AD) mouse model of AD: (1) can a neural network learn features to discriminate the two groups consistently in a longitudinal period, and (2) can a neural network be trained to discriminate in time points outside the trained/learned period?

## Material and methods

### Ethics

This study was approved by the Animal Welfare Committee of the Coimbra Institute for Clinical and Biomedical Research (iCBR), Faculty of Medicine, University of Coimbra, and by Direção-Geral de Alimentação e Veterinária (DGAV). All procedures involving mice were conducted following the ARRIVE guidelines^17^ and also as per the Association for Research in Vision and Ophthalmology statement for animal use, and in agreement with the European Community Directive Guidelines for the care and use of nonhuman animals for scientific purposes (2010/63/EU), transposed into the Portuguese law in 2013 (DL113/2013). All animals were housed in a certified facility, with a temperature-controlled environment under a 12:12 h light–dark cycle, and food was provided ad libitum. All acquisitions were performed during the light phase. OCT acquisitions were performed under light anaesthesia with 80 mg/kg ketamine and 5 mg/kg xylazine (IP), the pupils dilated with 0.5% tropicamide and 2.5% phenylephrine, and ocular analgesia was achieved with oxibuprocaine.

### Data source and selection

**Figure 1:**
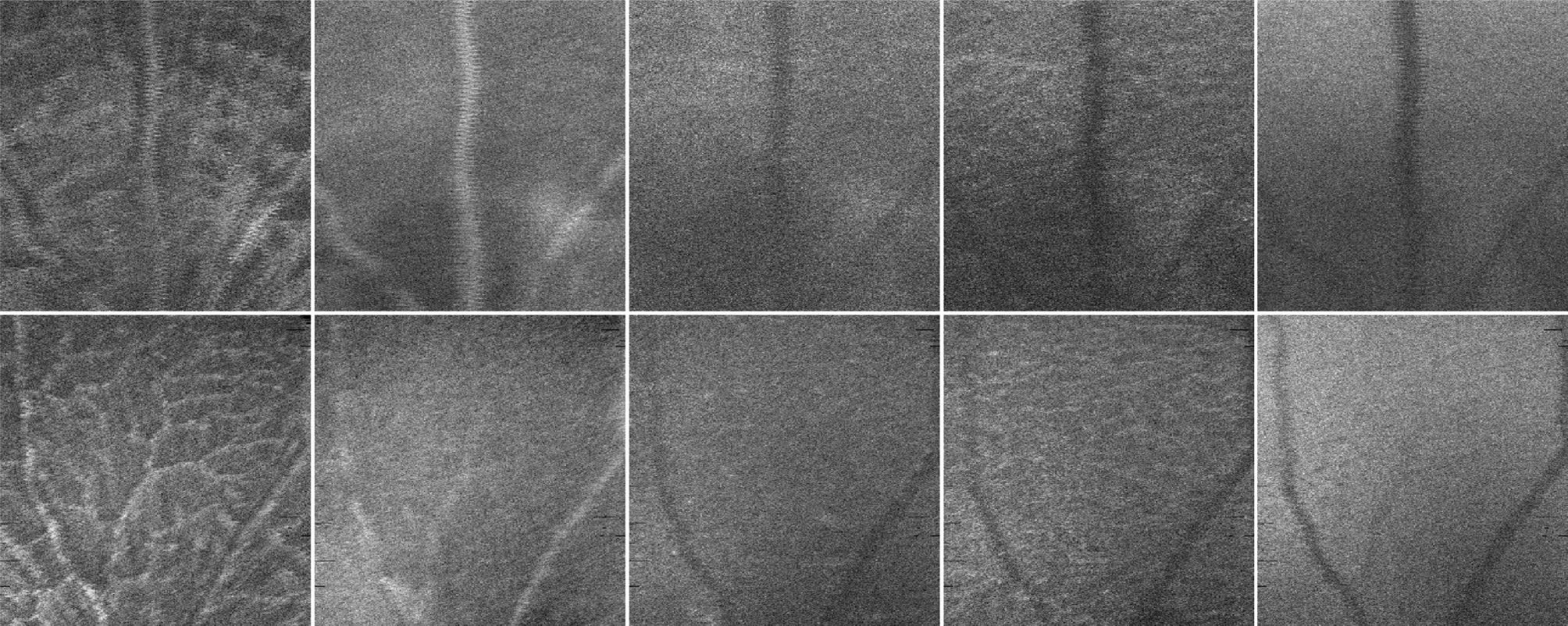
MVF (mean-value fundus) images examples from two left eyes of mice two months old (top: WT, bottom: 3xTg-AD). From left to right: RNFL-GCL (retinal nerve fibre layer-ganglion cell layer) complex, IPL (inner plexiform layer), INL (inner nuclear layer), OPL (outer plexiform layer), and ONL (outer nuclear layer).

The retinas of both eyes of wild type (WT) (C57BL6/129S) and 3xTg-AD mice were imaged using a Micron IV OCT System (Phoenix Technology Group, Pleasanton, CA, USA) at the ages of 11 (weight: 14.9 ± 2.6 g), 2 (weight: 22.4 ± 2 g), 3 (weight: 25.4 ± 2.1 g), 4 (weight: 27.2 ± 2.1 g), 8 (weight: 31.6 ± 3.4 g), and 12-months-old (weight: 33.5 ± 3.8 g). Volumes with insufficient image quality, low contrast, or leading to segmentation errors were excluded from the dataset. Also, from the initial 57 WT and 57 3xTg-AD mice, respectively, 7 and 13 deceased over the time window of the study (mice died between the ages of 1–2 (4 WT and 1 3xTg-A), 2–3 (1 3xTg-A), 3–4 (2 3xTg-A), 4–8 (2 WT and 4 3xTg-A) and 8–12 (1 WT and 5 3xTg-A)). For the remaining volumes, OCT data was segmented as disclosed in^16^ to compute five mean value fundus (MVF) images (Fig. 1) corresponding to retinal layers/layer-aggregate of the neuroretina: the retinal nerve fibre layer and ganglion cell layer complex (RNFL-GCL), the inner plexiform layer (IPL), the inner nuclear layer (INL), the outer plexiform layer (OPL), and the outer nuclear layer (ONL). In MVF images^18^, each pixel’s value is the average of the respective A-scan values within the boundaries of the layer of interest, therefore mimicking a fundus photograph if taken in the absence of all remaining layers.

The number of acquisitions per group, eye, and age used in the present study is presented in Table 1. Altogether, these yielded a total of 1144 MVF images per layer/layer-aggregate.

**Table 1:** Number of acquisitions (OCT volume scans) per group (WT–wild-type mice; 3xTg-AD–transgenic mice), eye (OD–right eye; OS–left eye), and age.

Mouse group	Eye	Age (months)					
		One	Two	Three	Four	Eight	Twelve
WT	OD	53	50	48	53	44	38
	OS	50	51	41	52	47	36
3xTg-AD	OD	44	51	52	49	46	40
	OS	52	52	54	52	46	43
Total	–	199	204	195	206	183	157

### Pre-processing and data augmentation

Because subtle differences may exist within the dataset between the two mouse groups, e.g. average intensity, intensity distribution, etc., all MVF images were normalised to ensure the same intensity distribution for all images, thus, limiting the possibility that discrimination is based on simple intensity-based metrics. First, we applied contrast-limited adaptive histogram equalisation (CLAHE) to adjust pixel intensities towards a uniform grayscale distribution and enhance local contrast. The resulting image was then low-pass filtered (LPF) (Gaussian filter) to find broader regional differences in intensity (filter parameters: 121 × 121 pixels’ kernel and standard deviation of 25). A new image resulting from the element-wise division of CLAHE by LPF was then computed and normalized to have zero mean and unit variance. Finally, this image’s variance was adjusted by multiplying it by 8 (ensuring all images are kept within the original range) and adding 128 (8-bit images).

Toward data augmentation, for each pre-processed image used for the training of the NN, two new images were created: one as the vertical flip (top–bottom flip), and another as a rotation of the original image of either 90° or 270°, being the angle chosen at random.

### Transfer learning from the inception-v3 model

While CNN models require substantial datasets for effective training, our database (per layer/layer-aggregate) consists of 1144 images only, yet to be split into training, validation, and test sets. The standard approach to overcome this obstacle is through the application of transfer learning, which was followed in this study by choosing the Inception-v3 network trained on the ImageNet dataset^19^ proposed by Szegedy^20^. Transfer learning was applied both via feature extraction and fine-tuning.

For the present application, the Inception-v3’s classification layer was replaced by a global average pooling layer, followed by a fully-connected layer with a sigmoid activation function to classify images as belonging to the WT or 3xTg-AD mouse group. Furthermore, only the weights of the last layer were updated during the training, and the fine-tuning was extended only to the last inception module.

The above procedure for transfer learning was independently repeated five times as an independent CNN was created for the RNFL-GCL complex, the IPL, INL, OPL, and ONL layers.

### Implementation

Data pre-processing was implemented in MATLAB Release 2021b (The Math-Works, Inc., Natick, Massachusetts, United States).

All neural network models were built, trained and verified in Python 3.7.9 using the Keras 2.7^21^ framework with TensorFlow 2.7^22^ as the backend. The network training was performed on a desktop, equipped with an AMD Ryzen 9 3900 × CPU @3.8 GHz with 12 cores, 64 GB RAM and an Nvidia RTX 3060 with 12 GB of memory, using CUDA version 11.5.

### Evaluation metrics

Performance metrics need to be chosen considering the problem being addressed and the distribution of the labels in the dataset. In binary classification tasks, like the present one, accuracy, sensitivity, specificity, and F1-score are commonly used to evaluate a model’s performance and its ability as a class predictor. Consequently, these were chosen for the present study.

### Training and testing

To address both questions put forward in the present study, data was split per animal to ensure no data from one animal can be present in more than one of the training, validation, and test sets. Also, and of the utmost importance, not all time points were considered for the training and validation sets. Specifically, only data from mice at the ages of 3, 4, and 8 months were considered for training and validation, leaving out data from 1, 2, and 12-month-old mice for testing purposes only. This division allows for testing the classification ability of the neural network on mice either younger or older than those used for training.

Data was split into train/validation and test sets, with 80% of mice being used in the train/validation set (46 WT and 46 3xTg-AD mice) and 20% used in the test set (11 WT and 11 3xTg-AD mice). Furthermore, a similar scheme was applied to split mice into the train (75%) and validation (25%) sets.

The number of eyes in the train/validation set is presented in Table 2; the test set is presented in Table 3.

**Table 2:** Train (T) and validation set (V): number of acquisitions (OCT volume scans) per group (WT–wild-type mice; 3xTg-AD–transgenic mice), eye (OD–right eye; OS–left eye), and age.

Mouse group	Eye	Age (months)					
		Three		Four		Eight	
		T	V	T	V	T	V
WT	OD	29	10	32	11	26	9
	OS	25	8	32	10	28	9
3xTg-AD	OD	32	10	29	9	28	9
	OS	32	11	31	10	29	10
Total	–	118	39	124	40	111	37

**Table 3:** Test set: number of acquisitions (OCT volume scans) per group (WT–wild-type mice; 3xTg-AD–transgenic mice), eye (OD–right eye; OS–left eye), and age.

Mouse group	Eye	Age (months)					
		One	Two	Three	Four	Eight	Twelve
WT	OD	10	9	9	10	9	8
	OS	9	9	8	10	10	7
3xTg-AD	OD	8	11	10	11	9	8
	OS	11	10	11	11	7	9
Total	–	38	39	38	42	35	32

Two independent tests were conducted, one testing only within the period considered for the training to answer the first scientific question (determine the feasibility of classification between WT and AD within the same age range), and one testing exclusively for ages outside of the training period to answer the second scientific question (determine the classification ability of the neural model outside its training age range).

## Results

Performance metrics were evaluated for the two tests detailed in the previous section. Results are shown in Table 4 (mice of 3, 4 and 8 months old in the training/validation/test sets) and Table 5 (mice of 3, 4 and 8 months old in the training and validation sets and mice of 1, 2 and 12 months old in the test set).

**Table 4:** Performance metrics for the classification into wild-type and the triple-transgenic mouse model of Alzheimer’s disease mouse groups, using 3, 4, and 8-month-old mice for the train, validation, and test sets; for the layer/layer-aggregates: the retinal nerve fibre layer and ganglion cell layer complex (RNFL-GCL), the inner plexiform layer (IPL), the inner nuclear layer (INL), the outer plexiform layer (OPL), and the outer nuclear layer (ONL).

Retinal layer	Accuracy	Sensitivity	Specificity	F1-score
RNFL-GCL	0.885	0.966	0.821	0.905
IPL	0.913	1.000	0.821	0.922
INL	0.852	0.983	0.714	0.872
OPL	0.826	0.881	0.768	0.839
ONL	0.800	0.949	0.643	0.829

**Table 5:** Performance metrics for the classification into wild-type and the triple-transgenic mouse model of Alzheimer’s disease mouse groups, using 3, 4, and 8-month-old mice for the train and validation sets, and younger (1 and 2-months-old), and older (12-months-old) mice for the test set; for the layer/layer-aggregates: the retinal nerve fibre layer and ganglion cell layer complex (RNFL-GCL), the inner plexiform layer (IPL), the inner nuclear layer (INL), the outer plexiform layer (OPL), and the outer nuclear layer (ONL).

Retinal layer	Accuracy	Sensitivity	Specificity	F1-score
RNFL-GCL	0.881	0.877	0.885	0.885
IPL	0.835	0.825	0.846	0.839
INL	0.844	0.825	0.865	0.847
OPL	0.807	0.702	0.923	0.792
ONL	0.798	0.789	0.808	0.804

Overall, the accuracy is high for both test sets, showing the discrimination power of these neural networks to distinguish between WT and transgenic mice. Even more so as the training group incorporates retinas of distinct ages, hence forcing the network for each layer/aggregate to learn common features across this time window.

Expectedly, all metrics show a decrease in performance for the second scenario, in which the test set is outside the age range of the training set. Surprisingly, the decrease in performance can be considered modest despite the large differences in the age of the older animals in the training group (8-months-old) and testing group (12-months-old). The same applies to the younger group, despite the lower age difference.

As classification errors distribute evenly between left and right eyes, in Table 6 we present the distribution of classification errors by group and age.

**Table 6:** Classification errors (number of errors/number of cases) per time point and mouse group (WT–wild-type mice; 3xTg-AD–transgenic mice).

Retinal layer	Mouse group	Age (months)						Subtotal	Total
		One	Two	Three	Four	Eight	Twelve		
RNFL-GCL	WT	1/19 5.3%	3/18 16.7%	3/17 17.6%	4/20 20.0%	3/19 15.8%	2/15 13.3%	16/108 14.8%	27/224 (12.1%)
	3xTg-AD	3/19 15.8%	4/21 19.0%	2/21 9.5%	0/22 0.0%	0/16 0.0%	2/17 11.8%	11/116 9.5%	
IPL	WT	2/19 10.5%	3/18 16.7%	5/17 29.4%	3/20 15.0%	2/19 10.5%	3/15 20.0%	18/108 16.7%	33/224 (14.7%)
	3xTg-AD	11/19 57.9%	3/21 14.3%	0/21 0.0%	0/22 0.0%	0/16 0.0%	1/17 5.9%	15/116 12.9%	
INL	WT	4/19 21.1%	2/18 11.1%	4/17 23.5%	7/20 35.0%	5/19 26.3%	3/15 20.0%	25/108 12.0%	41/224 (16.7%)
	3xTg-AD	9/19 47.4%	3/21 14.3%	0/21 0.0%	0/22 0.0%	1/16 5.9%	3/17 17.6%	16/116 13.8%	
OPL	WT	1/19 5.3%	2/18 11.1%	4/17 23.5	6/20 30.0%	3/19 15.8%	1/15 6.7%	17/108 15.7%	47/224 (21.0%)
	3xTg-AD	15/19 78.9%	4/21 19.0%	1/21 4.8%	1/22 4.5%	5/16 31.3%	4/17 23.5%	30/116 25.9%	
ONL	WT	2/19 10.5%	6/18 33.3%	3/17 17.6%	10/20 50.0%	7/19 36.8%	3/15 20.0%	31/108 28.7%	51/224 (22.8%)
	3xTg-AD	12/19 63.2%	3/21 14.3%	1/21 4.8%	0/22 0.0%	2/16 12.5%	2/17 11.8%	20/116 17.2%	
Total	–	60/190 31.6%	33/195 16.9%	23/190 12.1%	31/210 14.8%	28/175 16.0%	24/160 15.0%	–	199/112 (17.8%)

Also, except for the RNFL-GCL complex, the classifications for the remaining layers present a significant portion of their errors at the age of 1-month-old. Indeed, this time point alone is responsible for 30% of the classification errors. Overall, the innermost layers of the retina present fewer errors, with these steadily increasing from the RNFL-GCL complex to the ONL.

Unsurprisingly, percentage-wise, fewer errors were found for the time points on which NNs were trained. On the other hand, most errors were found for the first time point, at the age of 1-month-old, while the error for the last time point is within the range of those for the three to 8-months-old. Indeed, the error at the 2-month-old time point is higher than that of 12-months-old despite the latter being further away from the training ages.

## Discussion

In this work, we used neural networks to classify computed fundus images from the retina of WT and 3xTg-AD mice from OCT data. This study did not intend to make associations with cell and molecular biology markers. Indeed, this mouse model of AD is well studied and documented in the literature from multiple viewpoints and assessed by numerous techniques analysing brain and retina functional, biochemical, biological and structural parameters^16,23–25^.

We kept all animals for the entire project timeframe in the present study instead of sacrificing some along the study period. This approach allowed us to perform the analysis herein that, otherwise, would require a much larger number of animals. A single model of the AD was considered as adding more would have rendered this study not feasible because of the number of animals involved. Nevertheless, this proof-of-concept can now be tested in other animal models of disease.

Instead of using the traditional approach of training and classifying cases of the same age or disease stage (a cross-sectional study), we aimed to perform this study across multiple ages to learn if a neural network can learn consistent features from cases at different stages of the natural progression, both on the ageing and disease progression. This is of crucial importance for AD since, in clinical practice, disease staging and duration is often unknown. Thus, understanding if a network can learn consistent features across multiple stages and ages is of relevance.

From the achieved performance in the first of the two scenarios (classification of test cases within the same age range of the training and validation tests), it is clear the capacity of all five networks to learn consistent features over time and use those correctly. Despite the high sensitivity in identifying 3xTg-AD mice across all retinal layers/layer-aggregates, the decrease in performance from the inner to the outer layers is evident from both the accuracy and F1-score, but above all from the specificity, which presents a significant decrease from the INL downwards. In consequence, these results suggest that the innermost layers offer more consistent characteristics over time and that these are specific for each of the considered mouse groups in the present study. Also, these results demonstrate the validity of the approach followed in assessing retinal changes due to the presence of human genes introduced in mice. However, prior studies of our research group using this animal model of Alzheimer’s disease have shown a reduction of significant differences in key metrics considered hallmarks of AD in the brain, especially in males, e.g., neural cell death, neuroinflammation, glial activity, etc.^23,24^. Still, the results of the present study show that substantial differences in the retina are present and detectable by our classification method.

Of paramount importance, though, is the demonstration of the possibility of achieving interesting classification results when classifying cases outside the training range, that is, younger and older mice than those in the training and validation sets. Indeed, layer-wise, similar results to the former scenario were found with the classification performance decreasing, in general, from the inner to the outer retinal layers. The first time point stands out from this analysis as it is responsible for 30% of the classification errors. This may be linked to the yet developing CNV (mice’s CNS increase in mass 4–15 weeks post-natal^26^), thus presenting more similar retinas, between groups, at this age, which would explain the much better performance achieved for the latter time point even though there is a significant gap in mice’ age comparing the oldest mice in both groups, 8 and 12-months-old, respectively in the training and validation groups. Interestingly, this would explain the better performance achieved in classifying mice of 12-months-old compared to the performance in classifying 2-month-old mice, despite the lower age difference to the training and validation sets. However, another possible explanation is that changes in the retina precede those in the brain, opening two possibilities. It suggests that introduced genes play a role in the neurodevelopment of the mouse model of AD, which would require revisiting prior studies and their conclusions or that the retina would be the right location to detect the early changes in the CNV associated with AD.

## Conclusions

In conclusion, the present work demonstrates the possibility of training a neural network to learn common features, across all ages, of the central nervous system and using those to identify cases outside the training range. This is of paramount importance as it puts forward the hypothesis of training a neural network based on existing data from patients and control groups and applying it to identify emerging cases or shed light on the changes unfolding in the human central nervous system. Furthermore, while our present study focuses on AD, this methodology might be extendable to other neurodegenerative diseases, following the same rationale that changes in the retina can be linked to changes in the brain, as both are part of the CNS.

## Data Availability

The dataset used in this article is available upon a formal and reasonable request from the corresponding author.

## References

[1] Jack CR, Lowe VJ, Weigand SD, Wiste HJ, Senjem ML, Knopman DS, Shiung MM, Gunter JL, Boeve BF, Kemp BJ, Weiner M, Petersen RC, Initiative ADN (2009). Serial PIB and MRI in normal, mild cognitive impairment and Alzheimer’s disease: Implications for sequence of pathological events in Alzheimer’s disease. Brain.

[2] 2020 Alzheimer’s disease facts and figures. (2020). *Alzheimer’s Dementia*. **16**(3), 391–460. 10.1002/alz.12068 10.1002/alz.12068 32157811

[3] Milletari F, Navab N, Ahmadi S-A (2016). V-Net: Fully convolutional neural networks for volumetric medical image segmentation. 2016 Fourth Int. Conf. Vision (3DV)..

[4] Sarvamangala DR, Kulkarni RV (2022). Convolutional neural networks in medical image understanding: A survey. Evol. Intel..

[5] Karthik R, Vaichole TS, Kulkarni SK, Yadav O, Khan F (2022). Eff2Net: An efficient channel attention-based convolutional neural network for skin disease classification. Biomed. Signal Process. Control.

[6] Yadav SS, Jadhav SM (2019). Deep convolutional neural network based medical image classification for disease diagnosis. J. Big Data.

[7] Chen Q, Hu S, Long P, Lu F, Shi Y, Li Y (2019). A transfer learning approach for malignant prostate lesion detection on multiparametric MRI. Technol. Cancer Res. Treat..

[8] Talo M, Yildirim O, Baloglu UB, Aydin G, Acharya UR (2019). Convolutional neural networks for multi-class brain disease detection using MRI images. Comput. Med. Imaging Graph..

[9] Shin H-C, Roth HR, Gao M, Lu L, Xu Z, Nogues I, Yao J, Mollura D, Summers RM (2016). Deep convolutional neural networks for computer-aided detection: CNN architectures, dataset characteristics and transfer learning. IEEE Trans. Med. Imaging.

[10] Zhang R, Zheng Y, Mak TWC, Yu R, Wong SH, Lau JYW, Poon CCY (2017). Automatic detection and classification of colorectal polyps by transferring low-level CNN features from nonmedical domain. IEEE J. Biomed. Health Inform..

[11] Yu X, Zeng N, Liu S, Zhang Y-D (2019). Utilization of DenseNet201 for diagnosis of breast abnormality. Mach. Vis. Appl..

[12] Zhu Y, Wang Q-C, Xu M-D, Zhang Z, Cheng J, Zhong Y-S, Zhang Y-Q, Chen W-F, Yao L-Q, Zhou P-H, Li Q-L (2019). Application of convolutional neural network in the diagnosis of the invasion depth of gastric cancer based on conventional endoscopy. Gastrointest. Endosc..

[13] Gómez-Valverde JJ, Antón A, Fatti G, Liefers B, Herranz A, Santos A, Sánchez CI, Ledesma-Carbayo MJ (2019). Automatic glaucoma classification using color fundus images based on convolutional neural networks and transfer learning. Biomed. Opt. Express.

[14] Byra M, Styczynski G, Szmigielski C, Kalinowski P, Michałowski Ł, Paluszkiewicz R, Ziarkiewicz-Wróblewska B, Zieniewicz K, Sobieraj P, Nowicki A (2018). Transfer learning with deep convolutional neural network for liver steatosis assessment in ultrasound images. Int. J. Comput. Assist. Radiol. Surg..

[15] Harper DJ, Augustin M, Lichtenegger A, Gesperger J, Himmel T, Muck M, Merkle CW, Eugui P, Kummer S, Woehrer A, Glösmann M, Baumann B (2020). Retinal analysis of a mouse model of Alzheimer’s disease with multicontrast optical coherence tomography. Neurophotonics.

[16] Ferreira H, Martins J, Moreira PI, Ambrósio AF, Castelo-Branco M, Serranho P, Bernardes R (2021). Longitudinal normative OCT retinal thickness data for wild-type mice, and characterization of changes in the 3×Tg-AD mice model of Alzheimer’s disease. Aging.

[17] Kilkenny C, Browne WJ, Cuthill IC, Emerson M, Altman DG (2010). Improving bioscience research reporting: The ARRIVE guidelines for reporting animal research. PLoS Biol..

[18] Guimarães P, Rodrigues P, Lobo C, Leal S, Figueira J, Serranho P, Bernardes R (2014). Ocular fundus reference images from optical coherence tomography. Comput. Med. Imaging Graph..

[19] Deng J, Dong W, Socher R, Li L-J, Li K, Fei-Fei L (2009). ImageNet: A large-scale hierarchical image database. IEEE Conf. Comput. Vision Pattern Recognit..

[20] Szegedy C, Vanhoucke V, Ioffe S, Shlens J, Wojna Z (2016). Rethinking the inception architecture for computer vision. IEEE Conf. Comput. Vision Pattern Recognit. (CVPR).

[21] Chollet, F. Keras. https://keras.io (2015)

[22] Abadi, M., Barham, P., Chen, J., Chen, Z., Davis, A., Dean, J., Devin, M., Ghemawat, S., Irving, G., Isard, M., Kudlur, M., Levenberg, J., Monga, R., Moore, S., Murray, D.G., Steiner, B., Tucker, P.A., Vasudevan, V., Warden, P., Wicke, M., Yu, Y., & Zhang, X. TensorFlow: A system for large-scale machine learning. in *Proc 12th USENIX Symp Oper Syst Des Implementation, OSDI 2016*, 265–283. (USENIX Association, 2016).

[23] Rodrigues-Neves AC, Carecho R, Correia SC, Carvalho C, Campos EJ, Baptista FI, Moreira PI, Ambrósio AF (2021). Retina and brain display early and differential molecular and cellular changes in the 3xTg-AD mouse model of alzheimer’s disease. Mol. Neurobiol..

[24] Chiquita S, Campos EJ, Castelhano J, Ribeiro M, Sereno J, Moreira PI, Castelo-Branco M, Ambrósio AF (2019). Retinal thinning of inner sub-layers is associated with cortical atrophy in a mouse model of Alzheimer’s disease: A longitudinal multimodal in vivo study. Alzheimer’s Res. Therapy.

[25] Chiquita S, Rodrigues-Neves AC, Baptista FI, Carecho R, Moreira PI, Castelo-Branco M, Ambrósio AF (2019). The retina as a window or mirror of the brain changes detected in alzheimer’s disease: Critical aspects to unravel. Mol. Neurobiol..

[26] Fu Y, Rusznák Z, Herculano-Houzel S, Watson C, Paxinos G (2013). Cellular composition characterizing postnatal development and maturation of the mouse brain and spinal cord. Brain Struct. Funct..

